# Development of a Self-Determination Theory-Based Physical Activity Intervention for Aged Care Workers: Protocol for the Activity for Well-being Program

**DOI:** 10.3389/fpubh.2018.00341

**Published:** 2018-11-26

**Authors:** Merilyn Lock, Dannielle Post, James Dollman, Gaynor Parfitt

**Affiliations:** Alliance for Research in Exercise, Nutrition and Activity (ARENA), School of Health Sciences, University of South Australia, Adelaide, SA, Australia

**Keywords:** physical activity, need support, affective valence, rating of perceived exertion, intervention mapping, aged care workers

## Abstract

Despite the well-established benefits of regular participation in physical activity, many Australians still fail to maintain sufficient levels. More self-determined types of motivation and more positive affect during activity have been found to be associated with the maintenance of physical activity behaviour over time. Need-supportive approaches to physical activity behaviour change have previously been shown to improve quality of motivation and psychological well-being. This paper outlines the development of a need-supportive, person-centred physical activity program for frontline aged-care workers. The program emphasises the use of self-determined methods of regulating activity intensity (affect, rating of perceived exertion and self-pacing) and is aimed at increasing physical activity behaviour and psychological well-being. The development process was undertaken in six steps using guidance from the Intervention Mapping framework: (i) an in-depth needs assessment (including qualitative interviews where information was gathered from members of the target population); (ii) formation of change objectives; (iii) selecting theory-informed and evidence-based intervention methods and planning their practical application; (iv) producing program components and materials; (v) planning program adoption and implementation, and (vi) planning for evaluation. The program is based in Self-Determination Theory (SDT) and provides tools and elements to support autonomy (the use of a collaboratively developed activity plan and participant choice in activity types), competence (action/coping planning, goal-setting and pedometers), and relatedness (the use of a motivational interviewing-inspired appointment and ongoing support in activity).

## Introduction

Regular participation in physical activity is known to be important for maintaining optimal physical and psychological well-being (1–4). However, despite the extensive body of scientific literature that supports the role of physical activity for health, and the large temporal and financial investments in health promotion programs that have accumulated over the past few decades, nearly 45% of Australians still fail to maintain recommended levels (5). These statistics indicate the need to find ways to better engage people in regular physical activity participation and to support long-term maintenance of physical activity behaviour.

Exercise-related affect (the emotional response associated with the behaviour) has been found to predict future physical activity behaviour, functioning in what has been termed the exercise-affect-adherence pathway (6–8). It is thought that exercise-related affect has an important relationship with an individual's motivation to exercise, operating as feedback to reinforce the behavior (9). From an evolutionary perspective, our species has evolved in an environment where physical activity was often necessary for survival or reproductive success, but the maintenance of energy balance relied on minimising unnecessary effort (10). It has been proposed that the homogenously negative affect associated with exercise intensities exceeding the ventilatory threshold may have evolved to prevent unnecessary exertion and to maintain energy balance (8, 11). Affective responses to activity undertaken at intensities around the ventilatory threshold are less consistent, possibly due to a greater influence of cognitive processes when physiological cues (i.e., heart rate and body temperature) are challenging homeostasis (the Dual-Mode Theory) (11–13). More positive affective responses, measured during exercise, have been associated with more positive attitudes, greater levels of self-efficacy, intentions to exercise and physical activity behaviour across time (6, 7, 9, 14).

Exercise-related affect seems to be related to future activity behaviour, but how can we use this knowledge to optimise affective valence? There are several methods that can be used to regulate physical activity intensity for the purpose of exercise prescription. Traditional methods generally include heart rate measures at intensities that correlate to a percentage of maximal heart rate, maximal oxygen uptake (VO_2max_), and heart rate or VO_2_ reserve (15). However, there is growing evidence that exercise-related affect, as measured by tools such as the Feeling Scale developed by Hardy and Rejeski (16), may provide a valid method of regulating activity intensity (17, 18). Affect-regulation for activity intensity may provide a means of optimising exercise-related affect to avoid negative affect and promote more positive affective valence. Exercise-related affect elicited at higher intensities may be perceived as more negative and may experience a greater rate of decline above the ventilatory threshold in women who have low levels of physical activity participation compared to those who are regularly active (19, 20). Similarly, the positive outcomes associated with purposely using affect-regulated activity to impact physical activity behaviour may be more pronounced in individuals with a lower level of cardiorespiratory fitness compared to their fitter counterparts (21). In this case the use of affect to regulate physical activity intensity may be particularly useful for those initiating an exercise program.

Another method of regulating activity intensity that has steadily established itself as a valid and practical tool is the Rating of Perceived Exertion (RPE), originally proposed by Gunnar Borg (22). While RPE has a much closer association with more traditional methods of regulating activity (i.e., as heart rate and oxygen uptake increase, so too does RPE), it may also provide a greater sense of participant control compared to tradition methods. Exercise undertaken at RPE 13 (“somewhat hard”) on Borg's 6–20-point scale sits within the range of moderate physical activity according to the American College of Sports Medicine (ACSM) definition (23). This intensity seems to be perceived as positive in previously sedentary participants, and still promotes cardiorespiratory benefits generally associated with traditionally prescribed moderate intensity exercise (24). Additionally, exercise prescribed at RPE 13 may have a positive impact on affect, adherence and motivational constructs when compared to activity prescribed at higher intensities such as RPE 15 and RPE 17 (19, 25). Therefore, when used strategically, RPE may be used to promote positive affect and improve cardiorespiratory fitness, while also being adaptable for more active participants and specific exercise prescription.

A third method of regulating activity intensity that has found increasing interest in the field of physical activity behaviour change is the recommendation of self-selected or preferred pace for activity. There is growing evidence which suggests that supporting an individual's control over exercise mode and intensity may improve the affective valence associated with the activity (26–28). Even small increases in intensity above preferred levels may have a negative impact on the affective response to the activity (29). Similarly, participation in an exercise mode that is least preferred has been found to elicit higher levels of perceived exertion, fatigue and psychological distress and lower measures of positive well-being compared to participation in a high-preference exercise mode (26, 30). A pilot study by Williams et al. (31) recommended self-paced walking in a group of low-active, overweight women and compared this to walking at 64–76% of maximum heart rate for a period of 6 months. The researchers found a number of non-significant trends including a lower mean exercise intensity in the self-paced group (58.7 vs. 62.0% of maximum heart rate). Despite this, the self-paced group completed more minutes per week of walking and had greater exercise energy expenditure equivalent to 26 min and 83 kilocalories per week, respectively. Much of the evidence shows a large inter-subject variability in self-selected pace but it seems that many people, including previously sedentary participants, are still likely to self-select an intensity that fits within the range recommended by the ACSM to obtain health benefits (32, 33). While the impact of self-paced exercise on health and fitness variables has not been empirically tested, a review by Ekkekakis and Panteleimon (33) found several trials where self-paced intensity showed heart rate values that fitted within the ACSM's definition of moderate exercise intensity. Furthermore, another review by Williams (34) posed a strong case for long-term health benefits of self-paced exercise through greater adherence compared to prescribed moderate intensity exercise. Overall, there is now a strong case supporting the use of alternative methods of regulating exercise intensity, such as affect, RPE, and self-pacing, for the support of behaviour and motivational processes (15).

*Self-Determination Theory (SDT)* is a theoretical framework that may support the positive impact of affect-, RPE- and self-paced regulation of exercise intensity on physical activity behaviour. The application of SDT within physical activity interventions may potentially improve exercise-related affect and promote long-term maintenance of physical activity behaviour change (35–39). One sub-theory within SDT, *Basic Psychological Needs Theory*, proposes that humans have innate psychological needs for autonomy, competence and relatedness that must be supported for optimal psychological well-being (40–42). The application of these “self-determined” methods of regulating activity intensity fits well within this framework as supporting the needs of autonomy and competence. There is some evidence that the use of a need supportive approach in physical activity-based interventions may improve psychological well-being (43, 44), the exercise-related affect (35), and the maintenance of physical activity behavior (45, 46).

It is now well-accepted that behaviour change techniques such as the use of self-monitoring and action planning can support self-efficacy and positively impact physical activity behaviour (47, 48). Trials that have incorporated self-management approaches (by their nature, supporting the needs of autonomy and competence) have also been successful at increasing physical activity behaviour in some clinical populations (49–54), and achieving improvements in psychological distress including depressive symptoms and anxiety (49, 55). The needs for autonomy and relatedness may be supported through the use of a collaborative and empathetic approach. Similar approaches, such as that used in Motivational Interviewing, have been successful in promoting behaviour change and fit well within the framework of SDT (56, 57).

Another sub-theory of SDT, *Organismic Integration Theory*, proposes that the regulation of behaviour in humans (motivations) sits along a continuum from less to more internalised (self-determined) regulation (38, 42, 58). An example of this would be the engagement in a behaviour for enjoyment or because it aligns with an individual's personal values. This behavioural regulation would be much more self-determined than engagement in the behaviour for monetary reward or fear of punishment. Past studies have found more self-determined forms of behavioural regulation to be a predictor of behavioural maintenance including self-monitoring, weight loss, and physical activity participation (45, 59–62), and more positive exercise-related affect (63, 64). The internalisation of behavioural regulation may also be facilitated through the use of a need-supportive approach (43, 65, 66). All of this information together emphasises the complex and bi-directional relationship that seems to exist between affect, motivation and behaviour.

Acknowledging the information outlined hereto, we propose a novel workplace physical activity program that is based in SDT (the Activity for Well-being program). This program aims to positively influence behavioural regulations, psychological well-being and promote longer-term maintenance of physical activity behaviour through the use of activity choice (mode and intensity), support of self-management, and emphasis on the use of self-determined modes of regulating exercise intensity (affect-regulation, self-pacing or RPE). This is a novel approach that is consistent with SDT and is supported by emerging research regarding the relationship between exercise, affect and adherence (24, 31, 34, 67). This paper outlines the systematic development of the Activity for Well-being Program using the Intervention Mapping framework. It also outlines the protocol for the feasibility testing and evaluation of the Activity for Well-being program in frontline aged care workers. Ethical approval for this project has been obtained from the University of South Australia, Human Research Ethics Committee. Registration with the Australian and New Zealand Clinical Trials Registry (registration number: ACTRN12617001395325) can be found at https://www.anzctr.org.au/Trial/Registration/TrialReview.aspx?id=373693. Universal Trial Number (UTN): U1111-1202-3589.

## Methods

### Intervention mapping overview

The current intervention was developed using the Intervention Mapping framework proposed by Bartholomew, Parcel and Kok (68). The six steps undertaken as a part of the Intervention Mapping framework were to (i) undertake a needs assessment; (ii) prepare matrices of change and performance objectives; (iii) selecting theory-informed intervention methods and practical applications; (iv) produce program components and materials; (v) plan program adoption, implementation and sustainability; and (vi) plan for evaluation (69). The evaluation plan includes evaluation of behavioural and well-being outcomes, change and performance objectives, and an in-depth process evaluation (reach, adoption, fidelity, dose delivered, and dose received, maintenance, and context). The project will use a mixed-methods approach consisting of three primary phases: a pre-intervention qualitative needs assessment, a pre-post feasibility study, and a mixed-methodsevaluation of the program.

### Intervention mapping step 1: needs assessment

The Intervention Mapping approach emphasises a need for a linkage between the program developers, the implementers, and the end-users (68, 69). For the development of the Activity for Well-being program, the working group consisted primarily of the research team (program developers) who linked with both management of various departments within the funding organisation (implementers), and frontline aged care workers (end-users). The initial three components of the needs assessment have been completed and consisted of: (1) consultation with implementers within the funding organisation; (2) appraisal of the scientific literature regarding the health risk of the population of interest; and (3) qualitative interviews with frontline aged care workers. As a result of the first three components of the needs assessment, the Activity for Well-being program was designed to include an individual-level needs assessment for each participant. This will be delivered during the implementation of the program in the form of a motivational interviewing-inspired appointment. This appointment will be undertaken with an Exercise Physiologist and will be used to introduce need-support for the participant and to collaboratively develop an individual activity plan.

The initial consultation with implementers at the funding organisation identified physical health, activity, and psychological well-being as target areas within the organisation. Frontline community and residential care workers (those providing support for older adults in-home and in residential living facilities) were identified by the organisation as high-risk populations due to the nature of their occupation. Once these target issues and target population were identified, further investigation into previous scientific literature was undertaken to understand the difficulties facing this occupational group and those with similar demographics. Frontline employees in aged care in Australia tend to be an ageing and largely female population, with 86% female workers in aged care services (compared to 45% of workers in all industries) and 58% of workers aged 45 years and older (compared to 38% in all industries) during the 2010–11 period (70). Frontline aged care workers also tend to deal with high levels of job insecurity, shift work, and physical workloads. Musculoskeletal injury is commonly acknowledged as a significant issue within this population, with the broader category of Community and Personal Service Workers making up 27% of females in Australia who incurred a work-related injury during the 2013–2014 period (71). However, in addition to this, 4% of all work-related mental health claims in the 5 year period between 2008/09 and 2012/13 were from the sub-group Personal Carers and Assistants (72). This latter statistic was associated with a median claim payment of AU$12,000 and a median of 10 weeks of work absence.

The second stage of the needs assessment found that occupational factors such as job insecurity, shift work, long work hours, and high work stress were associated with a number of health issues in similar demographic and occupational groups including nurses, and residential and community-based care workers. These health issues include an increased prevalence of overweight and obesity (73–75), unhealthy behaviours such as smoking and physical inactivity (74, 75), increased risk of morbidity and mortality (76, 77), and depression and poor mental health (78, 79). Similarly, poor physical, and psychological health have been associated with low work ability (the perceived physical and psychological ability to cope with the demands of the occupation) (74, 80), decreased workplace productivity (81), and long-term sickness absence (74, 80, 82). Exacerbating these issues is the impact that ageing may have on employees in highly physical occupations. Previous studies have found that age-related reductions in physical work capacity and decreased musculoskeletal fitness can increase the risk of musculoskeletal injury, declining work ability, and retirement on disability pensions when paired with high physical workloads (83–86). There is some evidence however, that many of these concerns can be improved or attenuated by regular participation in physical activity (87–90).

The third stage of the needs assessment consisted of qualitative, semi-structured telephone interviews, undertaken with frontline aged care workers (*n* = 10; community-based support workers). These interviews identified a number of barriers to and enablers of volitional physical activity for these employees, along with perceived wants and needs from the program. The most common barrier for the sample group was a perceived lack of time, generally related to home and family commitments as competing priorities. Physical workload and fatigue, injury, scheduling issues, and motivational factors also played a significant role in decreasing physical activity participation. While ageing did not act as a barrier to physical activity participation in general, it did seem to impact activity choice, patterns, and motives. Enablers were widely varied between the individual participants, but notable enablers included making time, choosing low cost, and flexible activity options, and managing fatigue. The most common motives for activity were enjoyment and mind health benefits (i.e., the management of stress, depression, or anxiety). Social support was identified as a potential enabler for activity, however variability of work schedules was found to be a barrier to making social commitments. The information outlined here, which was gathered during these interviews, was used to inform the program design and the plan for implementation. The final step of the needs assessment was built into the program to occur at an individual participant level.

### Intervention mapping step 2: matrices of change objectives

**Figure 1:**
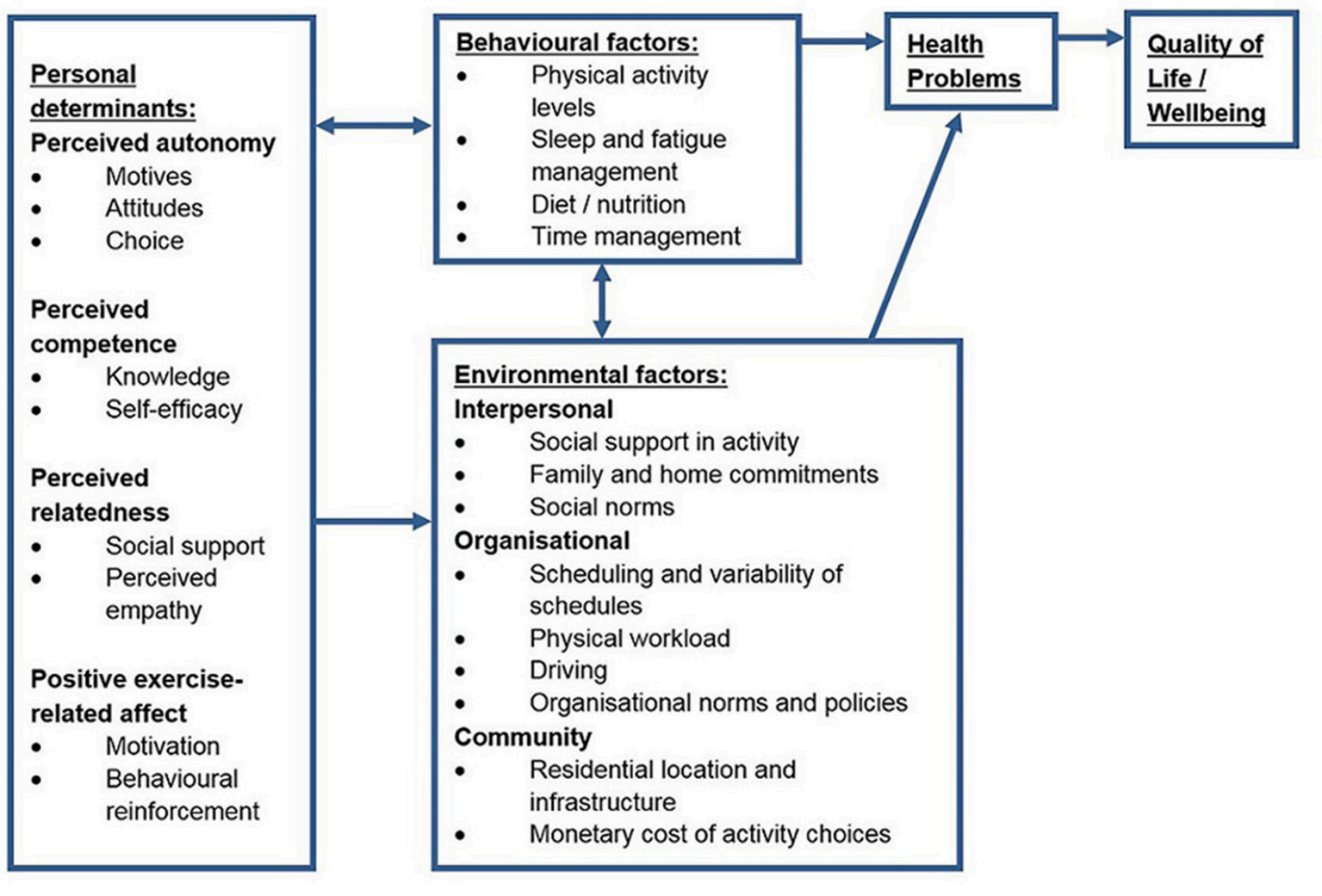
Logic model of the health issue.

The second step in the Intervention Mapping process involved the development of several matrices and a logic model to represent the health issue and the proposed methods of change. This step also involved selecting the target behavioural determinants (69). A number of specific behavioural determinants were identified both within the scientific literature and through the qualitative component of the needs assessment. The final changeable, target determinants were selected using a broader SDT-based approach which incorporated many of these specific determinants. The target behavioural determinants for the Activity for Well-being program included perceived autonomy, perceived competence, perceived relatedness, and positive exercise-related affect. Performance objectives were developed as “sub-behaviours” of the target behaviour. Three performance objectives were identified as important sub-behaviours for this population (find time to undertake physical activity, find motivation to undertake physical activity, and identify opportunities to undertake physical activity). Development of these performance objectives was informed by the qualitative component of the needs assessment. The resulting 12 change objectives were created as composites of the behavioural determinants and performance objectives. Incorporating information gathered from the needs assessment and the selected behavioural determinants, a logic model of the health issue was developed and is shown in Figure 1.

### Intervention mapping step 3: selecting theory-informed intervention methods and practical applications

**Figure 2:**
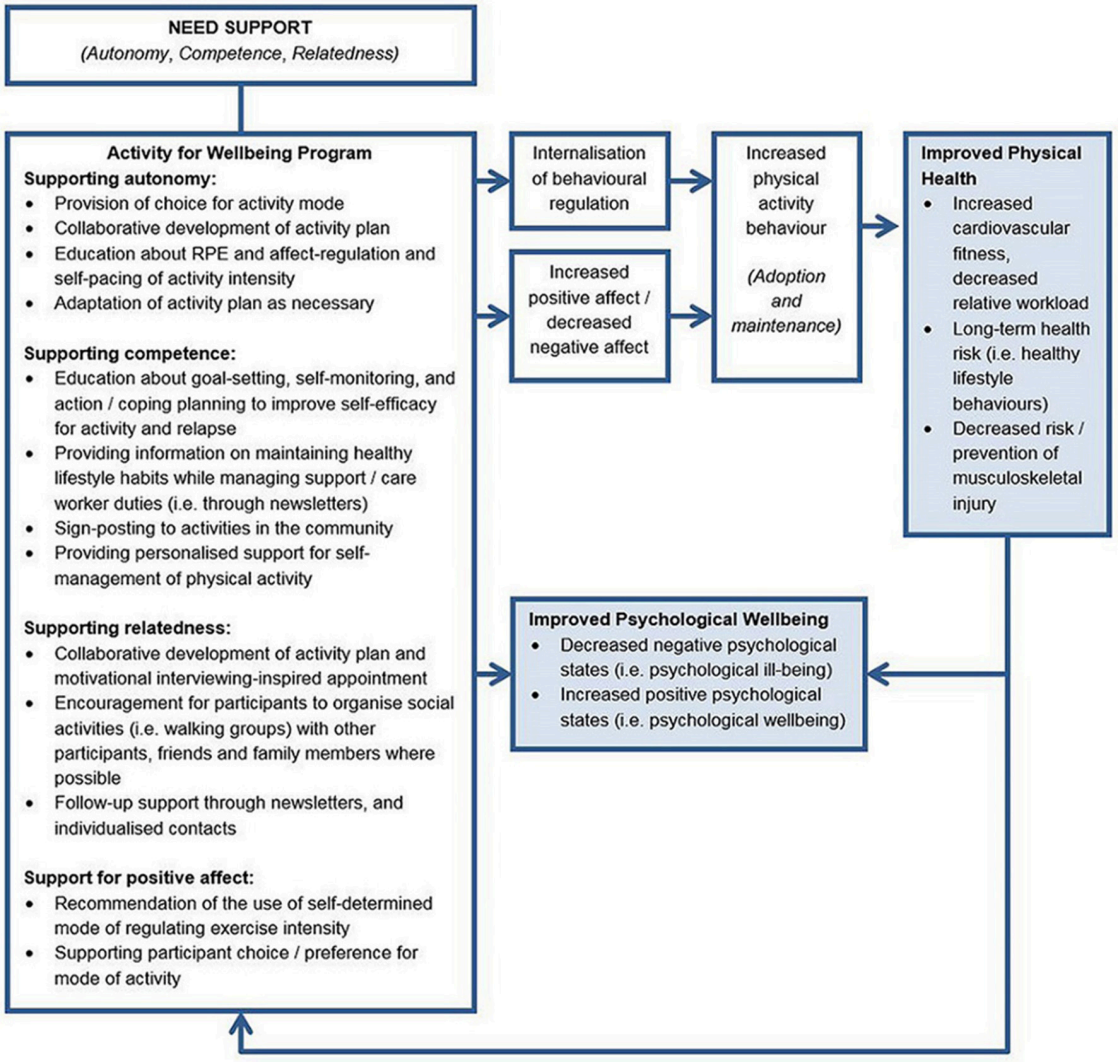
Change model of proposed intervention effects. RPE, Rating of Perceived Exertion.

The selection of theory-informed intervention methods and practical applications for the Activity for Well-being Program was undertaken concurrently with step 2 of the Intervention Mapping framework (developing change objective matrices). A need-supportive, Self Determination Theory-based approach that emphasised self-determined methods of regulating activity intensity was selected for the basis of the Activity for Well-being program. This approach is consistent with the background information that was outlined in the Introduction section of this paper and the outcomes of the needs assessment. Theory—and evidence-based practical applications of theory were systematically applied to each of the 12 change objectives. Most strategies were applied to multiple change objectives and some change objectives were addressed with multiple strategies. A change model for the proposed effects of the Activity for Well-being program is shown in Figure 2.

### Intervention mapping step 4: producing program components and materials—the activity for well-being program

As a result of the first three Intervention Mapping steps, the final program components were developed and are outlined here: Participants will undertake an initial need-supportive interview with an Exercise Physiologist (addressing six of 12 change objectives). The Motivational Interviewing-inspired interview will be undertaken using an empathetic and autonomy supportive approach and will use collaboration with the participant to develop an individualised activity plan and promote motivation for change (91, 92). During the course of the interview, the Exercise Physiologist will collaborate with the participant to develop an individualised and realistic physical activity plan that provides participant choice for activity. The participants will be encouraged to incorporate friends and family into individual activity plans to support environmental and social support outside of the participant-practitioner interactions. The Exercise Physiologist will provide a supporting role and may assist the participant to identify activities within the community (i.e., dance or exercise classes, social sporting groups) that he or she may want to participate in, develop a home, gym or walking program, and provide any other motivational, or technical support that the participant feels that he or she needs. The interview will be also used to obtain information regarding participant goals, exercise history and barriers to activity to guide this process. During this initial appointment the Exercise Physiologist will also educate the participant on goal-setting, action, and coping planning, and using affect, RPE and self-pacing for activity intensity (four of 12 change objectives). Each participant will have previous exposure to the use of Borg's RPE scale (22) and Hardy and Rajeski's Feeling Scale (16) while undertaking a 6 min walk during baseline testing.

Following the initial interview, participants will receive 3 months of individualised support for activity behaviour change (four of 12 change objectives). The support will include the use of self-monitoring tools, self-management strategies, monthly informational newsletters, and motivational messaging (directed at supporting autonomy, competence and relatedness) delivered through newsletters and individual contacts (via website, email, or SMS). All contacts from the Exercise Physiologist will aim to provide positive feedback on successes, while also providing empathy for challenges and reinforcing that relapses are normal and can be overcome. The Exercise Physiologist will facilitate group-based activities with work peers when requested by the participants and where possible. All participants will have access to pedometers for self-monitoring if desired and ongoing access to the program website. At the end of the 3 months, participants will continue to access self-monitoring tools and receive newsletters. The Exercise Physiologist will cease active follow-ups to promote autonomy, but will still continue to provide support for the following 6 months, when requested by the participants.

The tools for self-monitoring and self-management that will be available for participants have previously shown success for improving physical activity-related outcomes. These include pedometers (52, 53, 93); an interactive website (53, 94); and education in the use of goal setting, action and coping planning that will be offered as potential self-management strategies (49, 95, 96). The website that will be used for the Activity for Well-being program has been used previously with a population of cancer survivors and has been updated and adapted for the aged care worker population. The website has several pages with links to activities in the local community, information about physical activity and healthy eating, and a step log that can be used to track step counts and set “tiered” step goals (three goals per week based on whether the participant is feeling good, bad, or okay) (52). The website will also have a forum to allow online interaction between program participants. To assist with minimising cost, location and environmental barriers to gym-based activity, participants will also have access to gym facilities that are located across a number of sites provided by the implementing organisation and the university (one of 12 change objectives).

**Figure 3:**
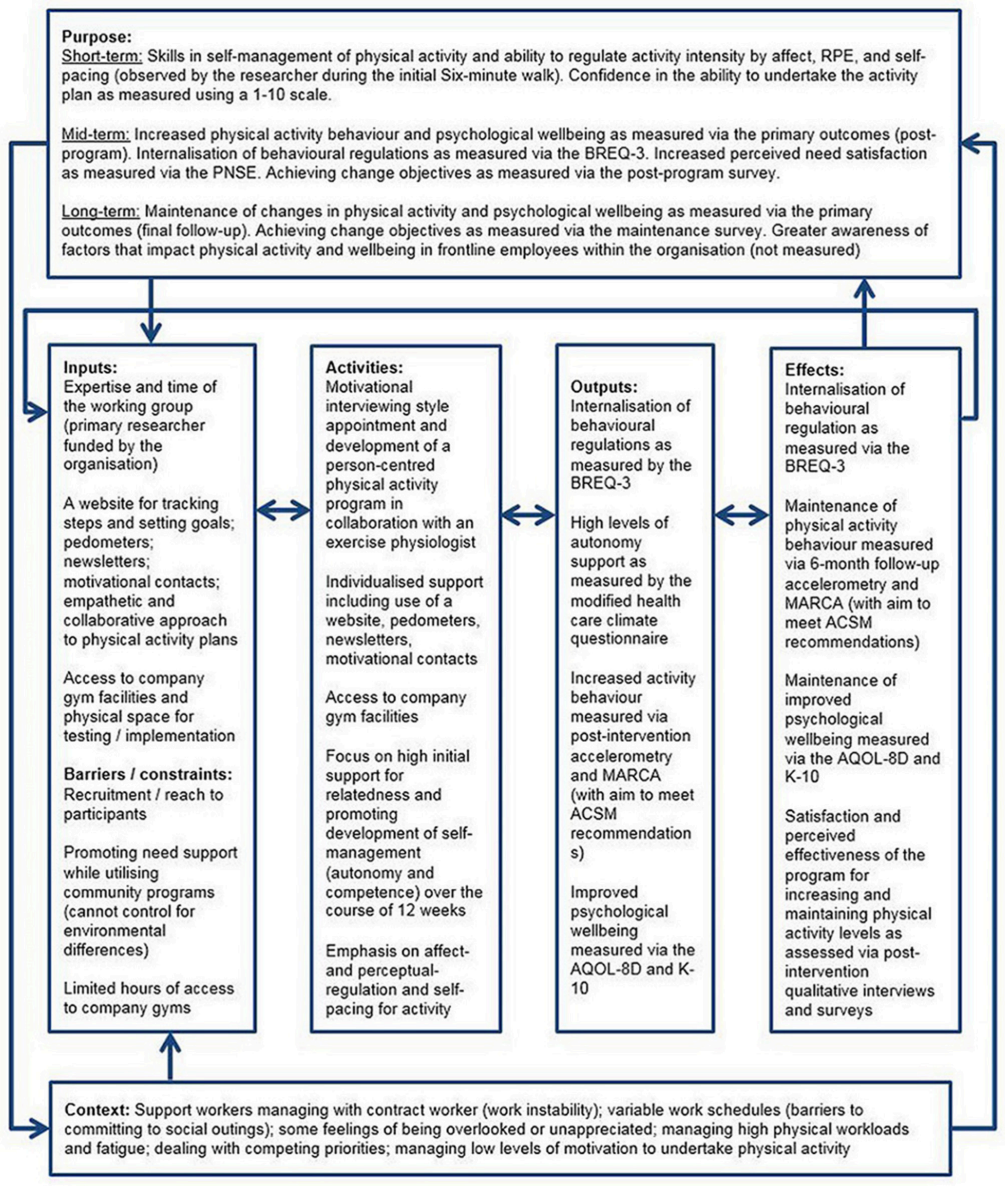
Logic model of the Activity for Well-being program. RPE, Rating of Perceived Exertion; BREQ-3, Behavioural Regulations in Exercise Questionnaire-3; PNSE, Perceived Need Support in Exercise questionnaire; MARCA, Multimedia Activity Recall for Children and Adolescents (adult-version); ACSM, American College of Sports Medicine; AQOL-8D, Assessment of Quality of Life-8D; K-10, Kessler 10-item Psychological Distress Scale.

While the pre-program needs assessment identified social support as a potential enabler, the need for flexible and individualised activity, due to variable work schedules and dispersed work locations (particularly for those based in the community), means that the activity program cannot be implemented as set activities in given locations or times. For this reason, the support for relatedness focuses on the interpersonal relationship between the Exercise Physiologist and the participant (through the motivational interviewing-inspired interview and individualised support). The Exercise Physiologist will also encourage participants to incorporate friends and family into their activity plan and will help to facilitate social activities with co-workers where possible. The final components of the Activity for Well-being program, and their application within the realm of SDT, are outlined in a logic model (Figure 3).

### Intervention mapping step 5: planning program adoption and implementation

#### Participants and research design

Participants will be self-selected from the population of frontline aged care workers, employed by the funding organisation. All participants will be recruited from the Adelaide metropolitan area and Fleurieu Peninsula, South Australia. Employees will be recruited via a generic email that will be sent out to all community-based support workers and care workers from participating residential sites. Expressions of interest will also be obtained through flyer drops and active promotion of the program at team meetings. This project will be undertaken using a mixed methods research design including a single-cohort, pre-post feasibility study, followed by a thorough process evaluation and qualitative evaluation of the program. Prior to commencing baseline testing, the researchers will obtain informed consent from each participant. Baseline testing will begin during the Australian late spring—early summer period and will be undertaken using a rolling recruitment until a sufficient number of participants have been enrolled in the program (~20–30 participants). Participants will complete a demographic questionnaire and undertake a pre-exercise screen using the Adult Pre-exercise Screening System (APSS) (97). The APSS is the Australian standard pre-exercise screening system that was developed as a collaboration between Exercise and Sports Science Australia, Fitness Australia and Sports Medicine Australia. Any participants who indicate previously diagnosed conditions, or signs/symptoms of cardiovascular disease in the first stage of the APSS will be required to obtain a medical clearance prior to undertaking exercise testing or program commencement. Participants will be included if they are 18 years of age or older, able to speak fluent English, currently employed as a frontline worker at the funding organisation and able to engage in moderate physical activity as defined by the American College of Sports Medicine (98). Ability to speak fluent English was placed as an inclusion criterion as many measures that will be used within this study are questionnaire-based and the lack of an ability to speak fluent English may impact the validity of these measures. Participants will receive an honorarium for undertaking the post-intervention and follow-up measures (a $25 and $50 gift card for these time-points, respectively). The honorarium will not be impacted by adherence to the intervention; these will be given for undertaking the measures only. As this is a feasibility study, sustainability of the program at an organisational level will not be addressed within the scope of this project; however, at an individual level the program was developed to promote long-term maintenance of behaviour change.

### Intervention mapping step 6: planning for evaluation

#### Process evaluation

##### Reach

Reach will be evaluated through a three-question online questionnaire that will be sent by email to all invited support workers and residential care workers at the end of the intervention period (after all participants have completed the active phase of the intervention). The survey will investigate the level of awareness of the program and the reasons why employees chose to participate in the study or not. As a part of the reach evaluation, baseline demographic data from program participants will also be compared to de-identified data of the whole target population to assess the representativeness of the sample. Data used for the evaluation of reach will be the mean (or median) age, proportion of males to females, and proportion of the sample based in each area (North, South, East, or West) or at each residential site.

##### Adoption

As the Activity for Well-being program has the support of higher-level management and it is expected the program will be adopted in all areas or sites that it is offered. Adoption will therefore be assessed as the level of perceived support of the program from implementing (i.e., management) staff. This will be evaluated by a seven-question, post-program, team leader survey. This short questionnaire will be sent to team leaders, site co-ordinators, and other implementation staff at the end of the intervention period and will survey the perceived aims, value, and observable impact of the program. It will also investigate the perceived barriers to and incentives for participation from the perspective of the implementing staff, and elements of maintenance at an organisational level (i.e., perceived value of maintaining a program such as this and intention to encourage the maintenance of healthy behaviours). Areas and sites that demonstrate high and low levels of perceived value and engagement with the program, type of service (i.e., residential or community), and geographical locations of sites and areas will be compared for differences in outcomes or recruitment numbers.

##### Fidelity

The fidelity of the implementation of the program (i.e., maintaining an autonomy supportive and collaborative approach) will be evaluated by audio-recording of a small number of randomly selected initial appointments. Recordings will be evaluated for fidelity by members of the research team who are not responsible for undertaking initial appointments with the participants, using a customised fidelity checklist. The checklist provides a score of “achieved” (1) or “not achieved” (0) for the fidelity of the interview based on three primary sections:

- Were all components (goal setting, action and coping planning, pedometer, website, RPE/Feeling Scale/self-pacing) of the intervention explained to the participant appropriately?
- Was the activity plan developed collaboratively? (Used preferred activities; was perceived as achievable by the participant as measured on a 1–10 scale and adapted as necessary; and activities not imparted by the practitioner without participant engagement or consent).
- And, did the initial and total discussion adhere to the core principles of Motivational Interviewing? (expresses empathy; develops discrepancy between current behaviour and goals; roll with resistance; support self-efficacy; support autonomy).

Perceived autonomy support will be evaluated using the Health Care Climate Questionnaire (99) that will be sent out as an online survey, also managed by members of the research team who are not responsible for undertaking initial interviews.

##### Dose delivered and dose received

Detailed logs of support and communications for participants and information on website engagement will be kept and retrospectively evaluated for dose delivered and received. Dose delivered for physical activity and other program components (different methods for regulating activity intensity and different self-management tools) will be calculated from initial activity plans, and dose received will be scored using self-reported frequency of use from the Activity for Well-being Post-program Survey.

##### Context

The context of the environment in which the program is implemented will be evaluated through the post-program qualitative interviews. The participants will be purposefully selected to include participants with a diverse range of responses to questionnaires and differing outcomes. The interviews will explore factors that may have played a role in the effectiveness of the program (i.e., the perceived effectiveness of some tools or strategies over others, effectiveness of recruitment strategies, and general feedback about the program).

#### Evaluation of impact and outcomes

##### Behavioural outcome objectives—physical activity behavior

The behavioural outcome objectives for the Activity for Well-being project are to increase physical activity behaviour at 3 months and to maintain potential increases at 9 months post-intervention. Physical activity behaviour will be objectively measured using 7 day continuous accelerometry (GENEactiv, UK) at three time points (baseline, 3 months and 9 months). Two-day physical activity recall using the adult version of the Multimedia Activity Recall for Children and Adolescents (MARCA, Aus) will be used to assess changes in activity patterns and use of time between the three time points.

Esliger, Rowlands (100) demonstrated good validity (*r* = 0.97, *p* < 0.001), and intra- and inter-test reliability (Coefficient of Variation 1.8 and 2.4%, respectively) of GENEactiv accelerometers as measured using an Instron Multi-Axis Shaking Table. The authors also demonstrated that these accelerometers are comparable to portable gas analysis for measuring Metabolic Equivalents (METs) of activity achieving an area under the receiver operating characteristic curve (AUC) of 0.98, 0.91, and 0.91 during sedentary, moderate and vigorous, respectively, and high sensitivity and specificity for these activity categories (72–98%). The adult MARCA software has demonstrated good convergent validity for measuring physical activity levels (METs) compared to accelerometry, with Spearman coefficients (rho) at 0.72 (0.49–0.86), and very high test-retest reliability with intra-class correlation coefficients (ICCs) ranging from 0.99 to 1.00 (101).

##### Additional physical measures

Additional physical measures to be undertaken at each of the three time points will consist of a 6 min walk, body mass, resting blood pressure and heart rate. Height will be measured at baseline using a Tanita Leicester portable stadiometer. Body mass will be measured using a standard portable scale (Tanita UM-018). Resting blood pressure and heart rate will be measured on an Omran automatic blood pressure monitor (HEM-7121). The 6 min walk will be undertaken on a set walking course at each site. As data collection will need to occur across a number of different sites, the walking courses will vary between sites; however, where possible, the walk course for each individual will be kept consistent between time points (all courses ≥ 30 m in length). The 6 min walk will be undertaken according to the recommendations of the American Thoracic Society (102). The 6 min walk will be used to indicate change in physical capacity and to familiarise participants with the use of Borg's RPE scale (22) and Hardy and Rajeski's Feeling Scale (16). The 6 min walk has demonstrated high test-retest reliability (ICCs, 0.94–0.97) and although results were variable, the test has been positively correlated to other measures of exercise capacity (maximal oxygen consumption and maximum METs; r values ranging between 0.21 and 0.71) (103–105).

##### Well-being outcome objectives—psychological distress and quality of life

The well-being outcome objectives will be the improvement of psychological well-being at three-months, and maintenance of improvements at 9 months. Well-being will be measured using the Kessler 10-item Psychological Distress Scale (K-10) and the Assessment of Quality of Life-8D (AQoL-8 D) questionnaires. The K10 has demonstrated very good overall accuracy with an AUC of 0.90 (95% CI: 0.89 to 0.91) (106). In addition to strong validity data, the K10 has also demonstrated very good sensitivity and specificity for the identification of target disorders comparable to other measures such as the Global Health Questionnaire (GHQ) (106, 107). The AQoL-8D shows good internal consistency with Cronbach's alphas for the various dimensions ranging between 0.81 and 0.96, except for that of senses (Cronbach's alpha 0.52) (108, 109). The AQoL-8D has greater sensitivity than previous versions of the questionnaire in the domains of mental health (108).

##### Interpersonal environmental outcome objectives

The primary interpersonal environmental outcome objectives for the Activity for Well-being program are to increase perceived need-support at 3 months and maintain potential increases at 9 months through the use of a SDT -based approach. Perceived need-support will be measured via the Psychological Need Satisfaction in Exercise Scale (PNSE) which demonstrates good internal consistency with Cronbach's coefficient α ranging between 0.90 and 0.91 for all subscales, as tested in a population of undergraduate university students (110). The convergent validity of the PNSE compared to the subscales of Intrinsic Motivation Inventory and the Exercise Motivation Inventory-2 ranges between 0.32 and 0.65 and the discriminant validity ranges between-0.01 and 0.30 (110). Organisational environmental outcomes will be generally limited by the resources and scope of this trial.

##### Additional process measures

Behavioural regulations will be measured using the third version of the Behavioural Regulation in Exercise Questionnaire (BREQ-3). This BREQ questionnaire categorises physical activity behavioural regulation into external, introjected, identified, integrated, and intrinsic regulation plus amotivation, and has shown good factorial validity and reliability (111–114). The Cronbach's coefficient alpha (α) for the BREQ-2 subscales range between from 0.73 to 0.86 (112) and 0.86 for the additional integrated regulation subscale included in the BREQ-3 (115).

In addition to the BREQ-3, the Exercise Causality Orientation Scale (ECOS) will be an additional process measure (included at baseline only). Although the influence of exercise causality orientations has been widely under-researched, one study showed a positive association between autonomy orientation and both positive affect and more self-determined types of behavioural regulation (116). Therefore, the ECOS will be included as a process measure to provide information regarding the influence of exercise causality orientation on exercise choices, other psychological processes and outcomes. The ECOS has demonstrated good factorial and convergent validity, internal consistancy (Crobach's alphas 0.59–0.77) and test-retest reliability (ICCs 0.71–0.77) (117).

##### Evaluation of program performance, change and performance objectives

Specific questions addressing each of the change and performance objectives (developed as a part of the Intervention Mapping process) will be included in the post-program survey to evaluate the perceived impact of the program on these objectives. A general evaluation of the program will be undertaken using semi-structured individual interviews with program participants and implementing staff within the organisation. Individual interviews will be undertaken using an emergent-systematic approach to obtain detailed information about the perceived effectiveness, strengths and limitations of the program and its implementation.

#### Data processing and analysis

This project will use a variety of analytical methods, applying qualitative and quantitative techniques. The process evaluation will compare descriptive statistics of the sample population and groups within the sample to de-identified data from the support and care worker population from all participating sites and areas. Fidelity recordings of the sub-sample of initial interviews will be scored by members of the research team who are not responsible for undertaking initial interviews using a customised fidelity checklist. An overall score for each recording will be calculated as a proportion of the highest possible score, and descriptive statistics will be used to present overall fidelity of the initial interview sub-sample. Mean scores will be calculated for individual items on the HCCQ (inversing the score for item 13) to produce a mean score of overall autonomy support. Per protocol analysis will be undertaken using a random effects mixed-model to compare baseline, 3 month and 9 month time points for all well-being and behavioural outcome measures. Accelerometer data will be processed using the cut-points outlined by Esliger, Rowlands (100) to determine total minutes of sedentary, light, moderate and vigorous activity for each 7 day period of continuous accelerometry. Domain-specific means will be calculated for the PNSE and BREQ-3 and these will be analysed for differences in well-being or behavioural outcomes and movement between domains (i.e., movement toward more-self-determined types of motivation and increases in perceived support of psychological needs). Qualitative interviews undertaken with program participants and implementation staff will be audio recorded and transcribed. The transcripts will be coded by two independent researchers, and analysed using a structured thematic approach as described by Braun and Clarke (118). Conflicts will be resolved through discussion between the two researchers. A third researcher will be consulted in situations where conflicts cannot be resolved.

## Discussion

This research outlines the development of a person-centred, need-supportive physical activity program for frontline aged care workers. The program was developed using the Intervention Mapping framework which included a participatory approach to the program development. During the development process the research staff collaborated with implementing staff from within the funding organisation and potential end-users (frontline aged care employees). The use of a participatory approach for the development of the Activity for Well-being program aims to improve the efficacy and appeal of the program for the support and care worker population. The project also builds on the current evidence supporting the use of psychological need-support to facilitate motivation and psychological well-being (43–46). This is a novel program that encourages the use of self-determined modes of regulating physical activity intensity with an aim to improve the quality of motivation and psychological well-being.

The Intervention Mapping framework used to develop the Activity for Well-being program provides a systematic method of incorporating psychological theory, empirical evidence, and input from end-users to assist with the development of effective health promotion programs (68). A previous meta-analysis of workplace physical activity interventions indicates that incorporating psychological theory may increase the effectiveness of the program (119). Along with the use of an evidence-based and participatory approach, the Activity for Well-being program was developed with clear theoretical underpinnings and hypothesised causal pathways for behaviour change, which may lend itself to high levels of efficacy. Whatever the level of rigour that is used in the process of developing a behavioural change intervention, complex interventions (i.e., interventions involving multiple interacting components, including those with a tailored design) can be impacted by many factors (120). The Medical Research Council (UK) has previously identified the need for planning the evaluation of complex interventions and has provided comprehensive guidance around this (120–122). A thorough process evaluation was built into the current project to identify potential reasons underlying variability in effectiveness or unexpected results. The use of a mixed methods approach to evaluate the program, incorporating quantitative and qualitative research methods, should provide detailed and valuable information regarding the acceptability and implementation of the program. Additionally, the use of objective measures of physical activity will provide high-quality assessment of physical activity behaviour. The inclusion of multiple process measures, to assess motivational processes and need-support, will provide important insight into the mechanisms of change.

Despite the strengths outlined here, the current project has several weaknesses. As the project will be undertaken as a single-cohort, pre-post feasibility study, behavioural and psychological measures may be underpowered to detect significant change. The lack of a control group may limit the conclusions that can be drawn regarding the efficacy of the program and will mean that confounding factors, such as seasonal variation in physical activity behaviour, cannot be accounted for. The effectiveness of the program may also be influenced by the context of the occupation. The needs assessment revealed a desire for social support including greater interaction with co-workers (particularly in regards to community-based employees who often work independently). The needs assessment also revealed that the variability of work schedules and competing priorities may create a barrier to making social commitments. These factors may impact the implementation of social aspects of the program which would be important for the support of relatedness. The program will encourage participants to incorporate friends and family into individual activity plans, facilitate group-based activities where possible, and provide a forum on the program website to optimise social support outside of that provided by the Exercise Physiologist.

Lastly, the development of this program for a specific occupational group may impact the representativeness of the results compared to other occupational groups, populations or cultures. Cultural differences in the emphasis of workplace wellness, or the context in which these programs are implemented, may impact program participation and outcomes. For example, according to the World Health Organization Healthy Workplace Framework and Model (123), the emphasis of workplace wellness initiatives ranges from a focus on general workplace health and safety in some regions (i.e., Africa, the Middle East, Brazil, and the Eastern Mediterranean), to incorporating elements of individual lifestyle change in others (i.e., the United States of America and Canada). The current program fits well into this latter approach and therefore may be more feasible within regions with this emphasis. Frontline aged care workers, as an occupational group, also face a number of unique challenges within their work. As such any health promotion program implemented within this population may lead to distinctly different outcomes compared to other populations or occupational groups. Since the approach used in the current study is fundamentally collaborative and person-centred, it may be reasonable to think that the approach could be adapted to suit a variety of different populations and occupational groups; however this may require a detailed needs assessment prior to implementing such a program within future research or clinical situations (thereby adding to clinician or researcher burden and increasing costs associated with the implementation of the program).

The outcomes of this research may have significant practical implications for clinical practice. If the need-supportive approach used within this study has a positive impact on physical activity behaviour and psychological well-being, it may support a move toward a more autonomy-supportive approach within clinical and practical settings. Similarly, it may indicate that the “prescription” of physical activity using more self-determined modes of regulating intensity should be considered as a feasible option within clinical practice. Future research should consider testing a similar approach in a fully powered, randomised controlled trial. While there is a small but growing base of literature supporting the role of need-support and exercise-related affect in the maintenance of physical activity behaviour (7, 14, 45, 46, 124), the impact of recommending self-determined modes of regulating activity intensity to effect activity behaviour and motivation is not well-known. The current paper has outlined the potential mechanisms by which the use of self-determined modes of regulating activity intensity may effect change in activity behaviour, motivation, and psychological well-being as a part of a need-supportive approach. The subsequent project will aim to investigate this approach within a workplace situation, however further research should explore the impact of recommending self-determined modes of regulating physical activity intensity within a less-complex intervention.

## Summary

The Activity for Well-being program is a novel physical activity program that focusses on the use of an empathetic and collaborative approach to physical activity behaviour change and encourages the use of self-determined methods of regulating activity intensity. The program is based in SDT and uses evidence-based strategies to aid physical activity behaviour change. This project also investigates the impact of a need-supportive approach on motivation and psychological well-being as indicated by preliminary evidence from other physical activity studies (43, 65, 66). The in-depth evaluation to be undertaken will investigate the strengths, limitations and feasibility of the program, and the process.

## Ethics statement

This study was carried out in accordance with the recommendations of the National Statement on Ethical Conduct in Human Research, National Health and Medical Research Council, Australia with informed consent obtained from all subjects. All subjects gave written, or formally documented verbal, informed consent in accordance with the Declaration of Helsinki. The protocol was approved by the Human Research Ethics Committee, University of South Australia.

## Author contributions

Development of this paper was undertaken initially by ML with close assistance from DP, including the development of the logic models and the evaluation plan. DP undertook analysis of qualitative data associated with the needs assessment (independent of, but concurrent with ML to ensure rigor of the qualitative methods). JD and GP have overseen the entire project from initiation to present and have contributed to the research design and implementation of all aspects of the plan outlined within this paper. All authors have contributed to refining the final drafts of this manuscript.

### Conflict of interest statement

The authors declare that the research was conducted in the absence of any commercial or financial relationships that could be construed as a potential conflict of interest.
